# Long-term administration of recombinant canstatin prevents adverse cardiac remodeling after myocardial infarction

**DOI:** 10.1038/s41598-020-69736-y

**Published:** 2020-07-30

**Authors:** Akira Sugiyama, Rumi Ito, Muneyoshi Okada, Hideyuki Yamawaki

**Affiliations:** Laboratory of Veterinary Pharmacology, School of Veterinary Medicine, Kitasato University, Higashi 23 bancho 35-1, Towada City, Aomori 034-8628 Japan

**Keywords:** Pharmacology, Cardiovascular diseases, Acute coronary syndromes, Heart failure

## Abstract

Myocardial infarction (MI) still remains a leading cause of mortality throughout the world. An adverse cardiac remodeling, such as hypertrophy and fibrosis, in non-infarcted area leads to uncompensated heart failure with cardiac dysfunction. We previously demonstrated that canstatin, a C-terminus fragment of type IV collagen α2 chain, exerted anti-remodeling effect against isoproterenol-induced cardiac hypertrophy model rats. In the present study, we examined whether a long-term administration of recombinant canstatin exhibits a cardioprotective effect against the adverse cardiac remodeling in MI model rats. Left anterior descending artery of male Wistar rats was ligated and recombinant mouse canstatin (20 μg/kg/day) was intraperitoneally injected for 28 days. Long-term administration of canstatin improved survival rate and significantly inhibited left ventricular dilatation and dysfunction after MI. Canstatin significantly inhibited scar thinning in the infarcted area and significantly suppressed cardiac hypertrophy, nuclear translocation of nuclear factor of activated T-cells, interstitial fibrosis and increase of myofibroblasts in the non-infarcted area. Canstatin significantly inhibited transforming growth factor-β1-induced differentiation of rat cardiac fibroblasts into myofibroblasts. The present study for the first time demonstrated that long-term administration of recombinant canstatin exerts cardioprotective effects against adverse cardiac remodeling in MI model rats.

## Introduction

Myocardial infarction (MI), an ischemic heart disease, induced by occlusion of coronary artery remains a leading cause of mortality throughout the world^1,2^. Ischemic injury by coronary arterial occlusion evokes massive cardiomyocyte death, which in turn forms an infarcted area^3,4^. In the early phase of MI, cardiac fibroblasts contribute to repair the infarcted area through the activation of biological functions, such as proliferation, migration and differentiation into myofibroblasts^5^. Myofibroblasts characterized by an abundant expression of α-smooth muscle actin (α-SMA) play a pivotal role during scar formation in the infarcted area through the synthesis of structural extracellular matrix (ECM) proteins^3^. Although maturation of scar tissue is important to maintain cardiac structure and function^6^, insufficient scar formation causes scar thinning, cardiac dysfunction and/or cardiac rupture^3^. The infarct size is an important in prognosis and mortality in MI patients^7^. On the other hand, the degree of cardiac remodeling in non-infarcted ventricles is also crucial in the prognosis of MI^4^. An adaptive cardiac hypertrophy in the non-infarcted area compensates the reduction of cardiac contractility^8^. In the chronic phase of MI, an interstitial fibrosis in the non-infarcted area is mainly induced by activated myofibroblasts, which increases ventricular stiffness^9^. The interstitial fibrosis and pathological cardiac hypertrophy result in eccentric hypertrophy with left ventricular (LV) wall thinning and dilatation^4,9^. The scar thinning in the infarcted area and the adverse cardiac remodeling in the non-infarcted area finally lead to uncompensated heart failure with cardiac dysfunction^4^. To date, an effective therapy to achieve a remission of the pathological condition after MI has not been established.

Canstatin, a C-terminus fragment of type IV collagen α2 chain, was discovered as an endogenous anti-angiogenic and anti-tumor peptide^10^. We previously demonstrated that canstatin exerts various biological effects in cardiac cells. Canstatin inhibited hypoxia-induced apoptosis in H9c2 rat cardiomyoblasts^11^. Canstatin promoted proliferation and secretion of matrix metalloproteinase (MMP)-2 and MMP-9, and inhibited contractile activity in myofibroblasts derived from the infarcted area of MI model rats^12^. We also clarified that canstatin was highly expressed in normal myocardium, but decreased in the infarcted area of MI model rats^12,13^. Moreover, we recently reported that a long-term administration of recombinant mouse canstatin suppressed isoproterenol-induced cardiac hypertrophy and fibrosis^14^. However, the effects of canstatin-treatment on MI have not been clarified. In the present study, we examined whether the long-term administration of recombinant mouse canstatin exhibits a cardioprotective effect against the scar formation and adverse cardiac remodeling in MI model rats.

## Results

### Canstatin improves survival rate in MI model rats

**Figure 1:**
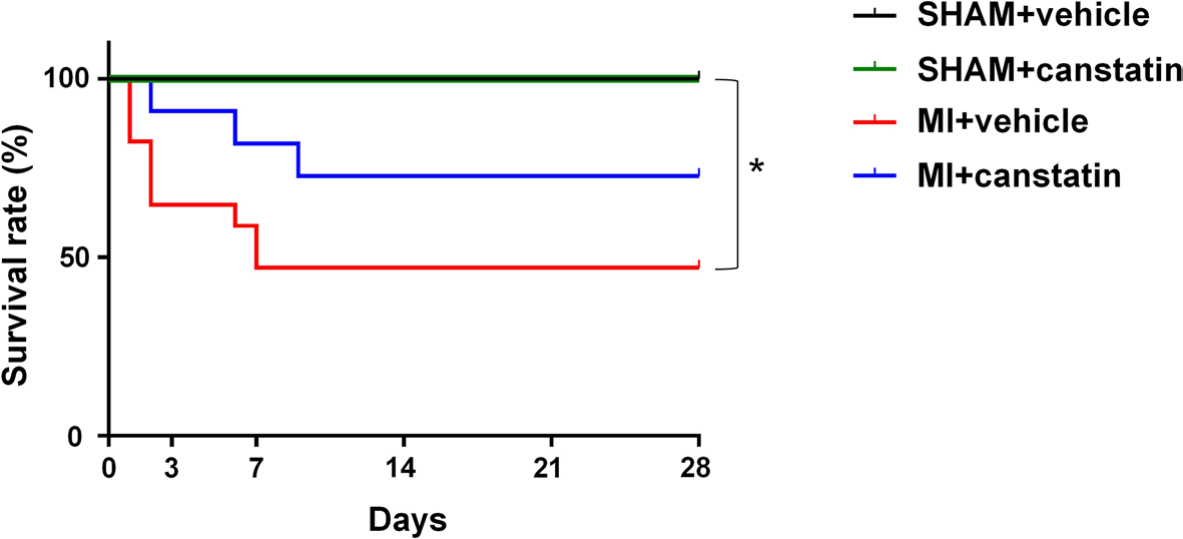
Canstatin improves survival rate after myocardial infarction (MI). MI was induced by ligating left anterior descending artery (LAD) in rats. Recombinant mouse canstatin (20 μg/kg) or its vehicle was intraperitoneally administered for 28 days after LAD ligation. Kaplan–Meier survival curves of SHAM-operated (SHAM) + vehicle (black; n = 6), SHAM + canstatin (green; n = 6), MI + vehicle (red; n = 17) and MI + canstatin (blue; n = 11) groups for 28 days were shown. *P < 0.05 vs. SHAM + vehicle (log-rank test).

All rats in SHAM-operated group (SHAM + vehicle and SHAM + canstatin groups) were survived for 28 days [SHAM + vehicle: 100% (6/6, n = 6), SHAM + canstatin: 100% (6/6, n = 6)]. MI significantly decreased the survival rate compared with SHAM + vehicle group [MI + vehicle: 47.1% (8/17, n = 17) vs. SHAM + vehicle, P < 0.05], and canstatin improved it (72.7%: 8/11, n = 11) (Fig. 1).

### Canstatin prevents LV dilatation and dysfunction in MI model rats

**Figure 2:**
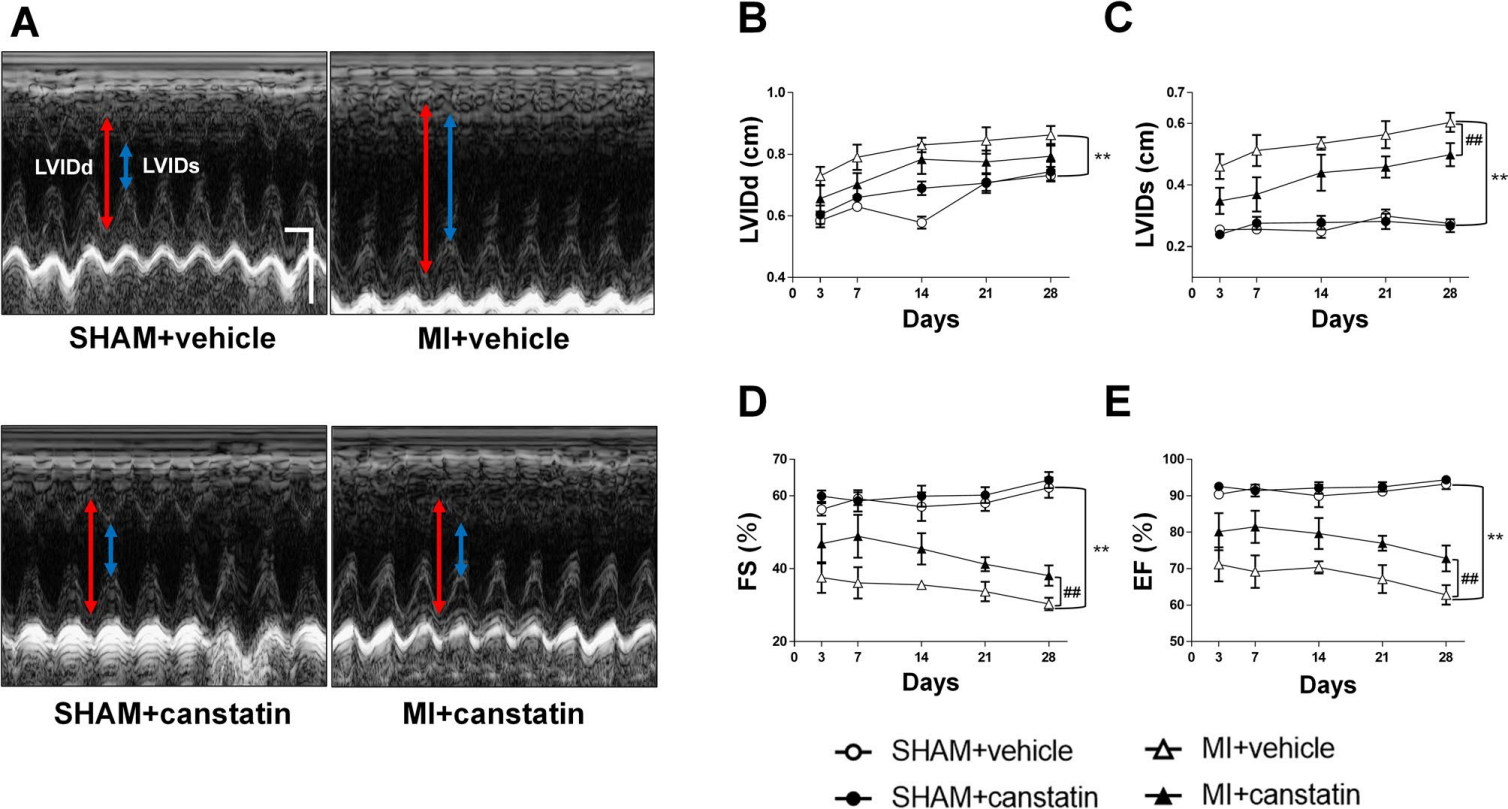
Canstatin prevents left ventricular (LV) dilatation and dysfunction after MI. Recombinant mouse canstatin (20 μg/kg) or vehicle was intraperitoneally administered for 28 days after LAD ligation in rats. Echocardiography was performed to analyze LV diameter and function. (**A**) Representative M-mode images from parasternal short-axis view of the LV in SHAM + vehicle, SHAM + canstatin, MI + vehicle and MI + canstatin groups were shown. Scale bar: 100 ms (horizontal) and 5 mm (vertical). LVIDd: left ventricular diameter at end-diastole (double headed red arrow). LVIDs: left ventricular diameter at end-systole (double headed blue arrow). Time course of LVIDd (**B**), LVIDs (**C**), fractional shortening (FS: **C**) and ejection fraction (EF: **D**) at 3, 7, 14, 21 and 28 days after MI was shown as mean ± standard error of the mean (S.E.M.). (SHAM + vehicle and SHAM + canstatin: n = 6; MI + vehicle: n = 8–17; MI + canstatin: n = 8–11). **P < 0.01 vs. SHAM + vehicle, ## P < 0.01 vs. MI + vehicle (two-way ANOVA followed by Tukey’s post hoc test).

We examined the effects of canstatin on LV diameter and cardiac function at 3, 7, 14, 21 and 28 days after MI by echocardiography (SHAM + vehicle and SHAM + canstatin: n = 6, MI + vehicle: n = 8–17, MI + canstatin: n = 8–11, Fig. 2A). The heart rate of rats was adjusted to approximately 400 beats per minutes by modulating the depth of anesthesia. MI significantly increased LV internal dimension at end-diastole (LVIDd) (P < 0.01, Fig. 2B) and LV internal dimension at end-systole (LVIDs) (P < 0.01, Fig. 2C). Administration of canstatin tended to inhibit the increase of LVIDd (Fig. 2B) and significantly inhibited the increase of LVIDs (P < 0.01, Fig. 2C). In addition, MI significantly decreased fractional shortening (FS) (P < 0.01, Fig. 2D) and ejection fraction (EF) (P < 0.01, Fig. 2E). Administration of canstatin significantly prevented the decrease of FS (P < 0.01, Fig. 2D) and EF (P < 0.01, Fig. 2E). Canstatin-alone administration had no effect on the LV diameter and cardiac function in the SHAM operated rats (Fig. 2B–E).

### Canstatin prevents scar thinning in MI model rats

**Figure 3:**
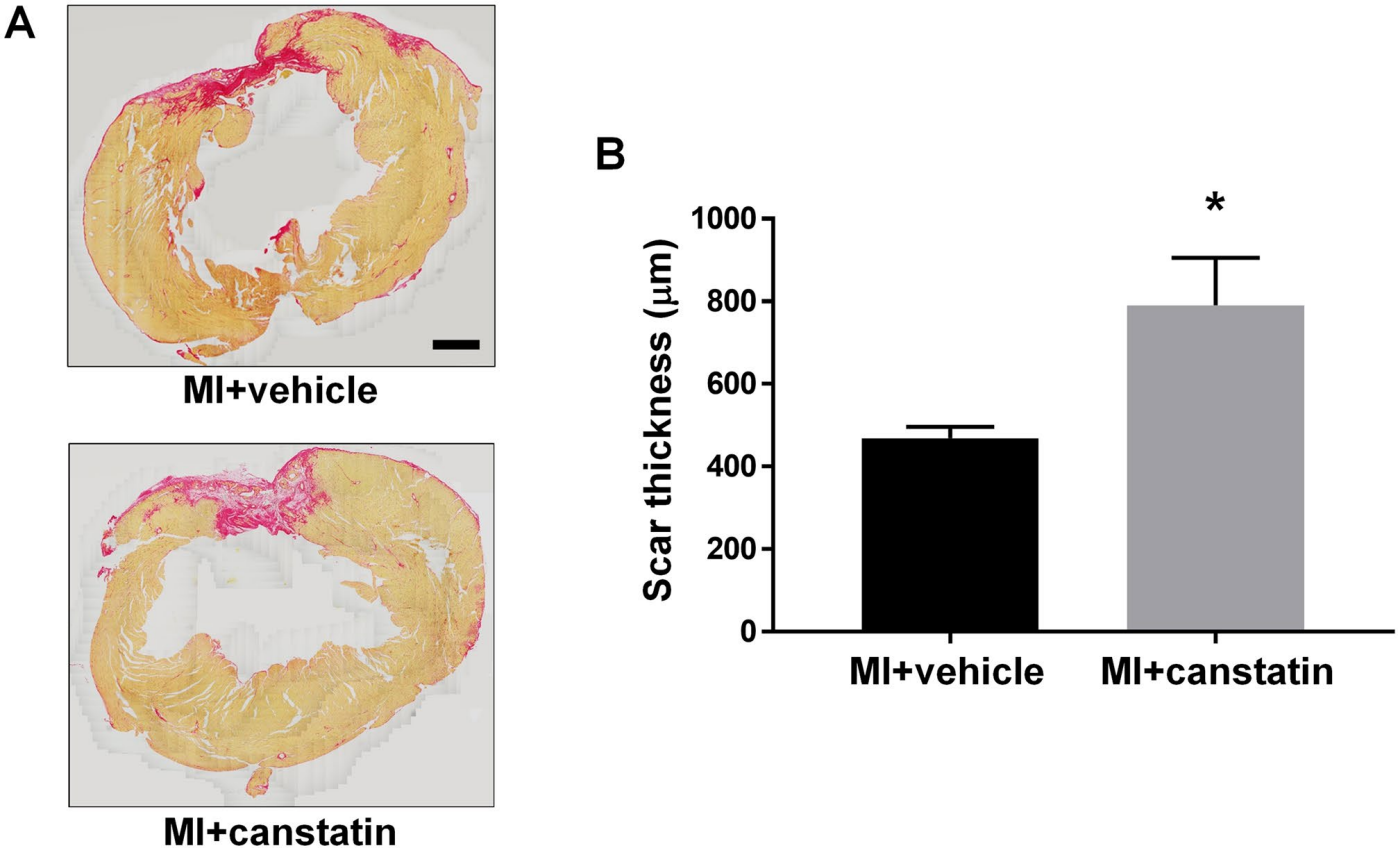
Canstatin promotes scar formation after MI. Recombinant mouse canstatin (20 μg/kg) or vehicle was intraperitoneally administered for 28 days after LAD ligation. The hearts were isolated and then thin paraffin Sects. (4 μm) were made. (**A**) Representative picrosirius red stained pictures of the LV from MI + vehicle and MI + canstatin groups were shown. Scale bar: 1 mm. (**B**) Scar thickness was measured and shown as mean ± S.E.M. (n = 8). *P < 0.05 vs. MI + vehicle (unpaired two-tailed Student’s t test).

Scar thinning after MI is a risk factor for lethal cardiac rupture^3,15^. We examined the effects of canstatin on scar formation by picrosirius red staining (MI + vehicle and MI + canstatin: n = 8, Fig. 3A). Administration of canstatin significantly improved the scar thinning after MI (MI + vehicle: 469 ± 27 μm vs. MI + canstatin: 791 ± 115 μm, P < 0.05, Fig. 3B).

### Canstatin inhibits cardiomyocyte hypertrophy of non-infarcted area in MI model rats

**Figure 4:**
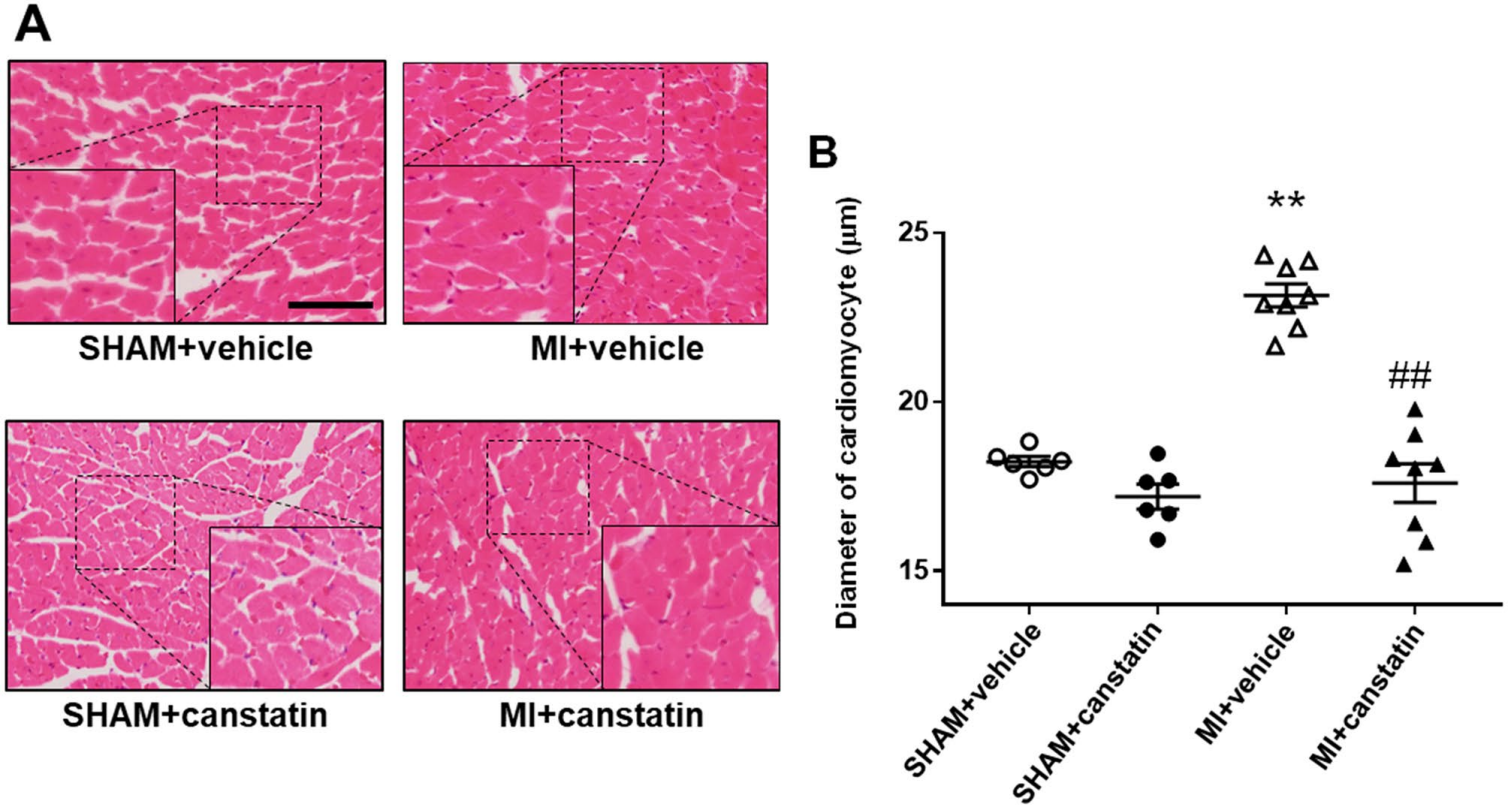
Canstatin prevents cardiomyocyte hypertrophy in non-infarcted area after MI. Recombinant mouse canstatin (20 μg/kg) or vehicle was intraperitoneally administered for 28 days after LAD ligation in rats. The hearts were isolated and then thin paraffin Sects. (4 μm) were made. (**A**) Representative hematoxylin and eosin stained pictures of the non-infarcted areas from SHAM + vehicle, SHAM + canstatin, MI + vehicle and MI + canstatin groups were shown. Scale bar: 100 μm. (**B**) Cross-sectional diameter of cardiomyocytes was measured (50 cells/heart) and shown as mean ± S.E.M. (SHAM + vehicle and SHAM + canstatin: n = 6; MI + vehicle and MI + canstatin: n = 8). **P < 0.01 vs. SHAM + vehicle, ## P < 0.01 vs. MI + vehicle (two-way ANOVA followed by Tukey’s post hoc test).

Cardiac hypertrophy of non-infarcted area was observed in the chronic phase of MI^4^. We examined the effects of canstatin on cardiac hypertrophy of non-infarcted area by measuring cross-sectional diameter of cardiomyocytes in LV using hematoxylin and eosin (HE) staining (SHAM + vehicle and SHAM + canstatin: n = 6, MI + vehicle and MI + canstatin: n = 8, Fig. 4A). MI significantly increased diameter of cardiomyocytes, which was significantly prevented by canstatin (MI + vehicle: 23.2 ± 0.3 μm vs. SHAM + vehicle: 18.2 ± 0.2 μm, P < 0.01; MI + canstatin: 17.6 ± 0.6 μm, P < 0.01 vs. MI + vehicle, Fig. 4B). Canstatin-alone administration had no effect on the diameter of cardiomyocytes (SHAM + canstatin: 17.2 ± 0.4 μm, Fig. 4B).

### Canstatin prevents nuclear translocation of nuclear factor of activated T-cells (NFAT)c4 in cardiomyocytes of non-infarcted area in MI model rats

**Figure 5:**
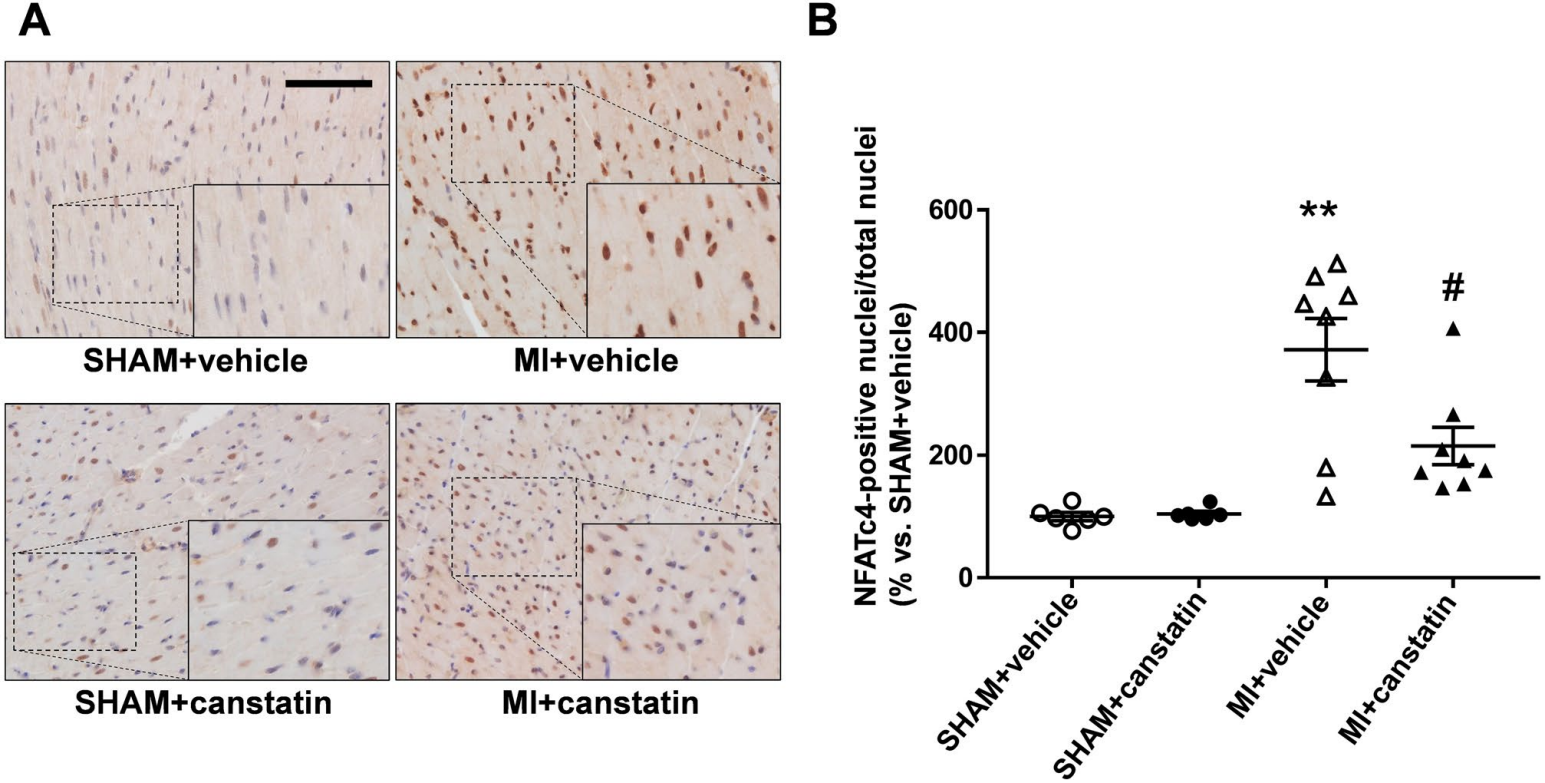
Canstatin prevents nuclear translocation of nuclear factor of activated T-cells (NFAT) c4 in non-infarcted area after MI. Recombinant mouse canstatin (20 μg/kg) or vehicle was intraperitoneally administered for 28 days after LAD ligation. The hearts were isolated and then thin paraffin Sects. (4 μm) were made. (**A**) Representative pictures of the non-infarcted areas from SHAM + vehicle, SHAM + canstatin, MI + vehicle and MI + canstatin groups reacted with a specific antibody against NFATc4 were shown. The nuclei were counterstained with hematoxylin. Scale bar: 100 μm. (**B**) The ratio of NFATc4-positive nuclei/total nuclei in 3 fields was counted, and the normalized ratio relative to SHAM + vehicle was shown as mean ± S.E.M. (SHAM + vehicle and SHAM + canstatin: n = 6; MI + vehicle and MI + canstatin: n = 8). **P < 0.01 vs. SHAM + vehicle, # P < 0.05 vs. MI + vehicle (two-way ANOVA followed by Tukey’s post hoc test).

Nuclear translocation of NFATc4 is associated with the transcription of hypertrophy-related genes in cardiomyocytes^16^. We previously reported that canstatin-administration inhibited nuclear translocation of NFATc4 in isoproterenol-induced cardiac hypertrophy model rats^14^. We then examined the effects of canstatin on nuclear translocation of NFATc4 in non-infarcted area by immunohistochemical staining (SHAM + vehicle and SHAM + canstatin: n = 6, MI + vehicle and MI + canstatin: n = 8, Fig. 5A). MI significantly induced nuclear translocation of NFATc4 in the cardiomyocytes of non-infarcted area, which was significantly prevented by canstatin (MI + vehicle: 372 ± 51% vs. SHAM + vehicle, P < 0.01; MI + canstatin: 215 ± 31%, P < 0.05 vs. MI + vehicle, Fig. 5B). Canstatin-alone administration had no effect on nuclear translocation of NFATc4 (SHAM + canstatin: 104 ± 4%, Fig. 5B).

### Canstatin prevents interstitial fibrosis of non-infarcted area in MI model rats

**Figure 6:**
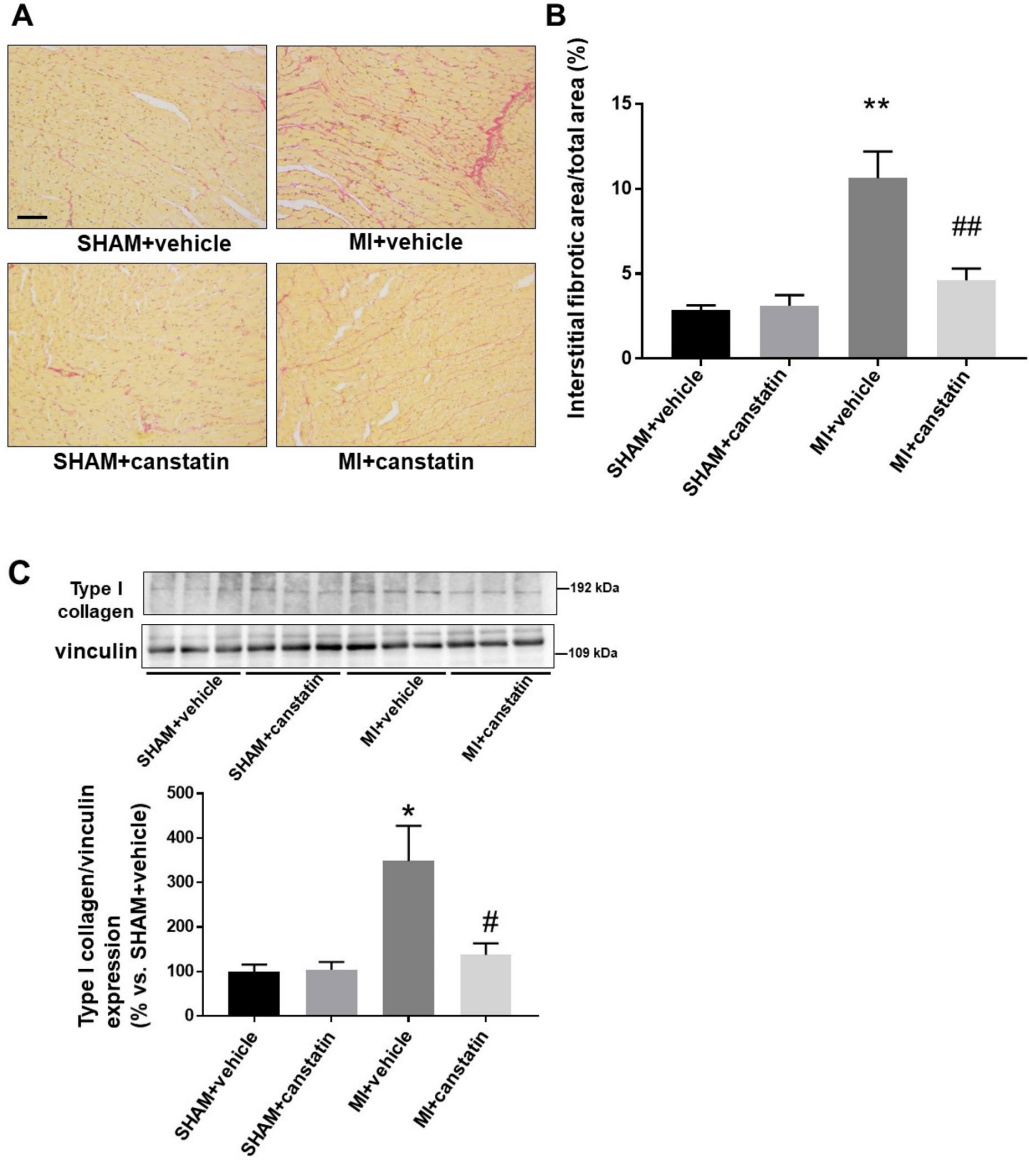
Canstatin prevents interstitial fibrosis in non-infarcted area after MI. Recombinant mouse canstatin (20 μg/kg) or vehicle was intraperitoneally administered for 28 days after LAD ligation. (**A, B**) The hearts were isolated and then thin paraffin Sects. (4 μm) were made. (**A**) Representative picrosirius red stained pictures of the non-infarcted areas from SHAM + vehicle, SHAM + canstatin, MI + vehicle and MI + canstatin groups were shown. Scale bar: 100 μm. (**B**) Interstitial fibrotic area/total area was measured and shown as mean ± S.E.M. (SHAM + vehicle and SHAM + canstatin: n = 6; MI + vehicle and MI + canstatin: n = 8). (**C**) The tissue protein of non-infarcted area was extracted. Expression of type I collagen was detected by Western blotting. (**Upper**) Representative blots of type I collagen and vinculin were shown. (**Lower**) Levels of type I collagen were corrected by vinculin and the normalized expression relative to SHAM + vehicle was shown as mean ± S.E.M. (SHAM + vehicle and SHAM + canstatin: n = 6; MI + vehicle and MI + canstatin: n = 8). *, **P < 0.05, 0.01 vs. SHAM + vehicle, #, ## P < 0.05, 0.01 vs. MI + vehicle (two-way ANOVA followed by Tukey’s post hoc test).

Interstitial fibrosis of non-infarcted myocardium causes cardiac dysfunction by increasing myocardial stiffness^9^. We examined the effects of canstatin on interstitial fibrosis of non-infarcted area by picrosirius red staining (SHAM + vehicle and SHAM + canstatin: n = 6, MI + vehicle and MI + canstatin: n = 8, Fig. 6A). MI significantly increased interstitial fibrosis area of non-infarcted area, which was significantly prevented by canstatin (MI + vehicle: 10.6 ± 1.6% vs. SHAM + vehicle: 2.9 ± 0.3%, P < 0.01; MI + canstatin: 4.6 ± 0.7%, P < 0.01 vs. MI + vehicle, Fig. 6B). Canstatin-alone administration had no effect on the interstitial fibrosis (SHAM + canstatin: 3.1 ± 0.6%, Fig. 6B).

We also examined the expression of type I collagen in non-infarcted area by Western blotting (SHAM + vehicle and SHAM + canstatin: n = 6, MI + vehicle and MI + canstatin: n = 8, Fig. 6C). MI increased the expression of type I collagen, which was significantly prevented by canstatin (MI + vehicle: 349 ± 74% vs. SHAM + vehicle: 100 ± 14%, P < 0.05; MI + canstatin: 137 ± 24%, P < 0.05 vs. MI + vehicle, Fig. 6C). Canstatin-alone administration had no effect on the expression of type I collagen (SHAM + canstatin: 104 ± 15%, Fig. 6C).

### Canstatin prevents differentiation of cardiac fibroblasts into myofibroblasts

**Figure 7:**
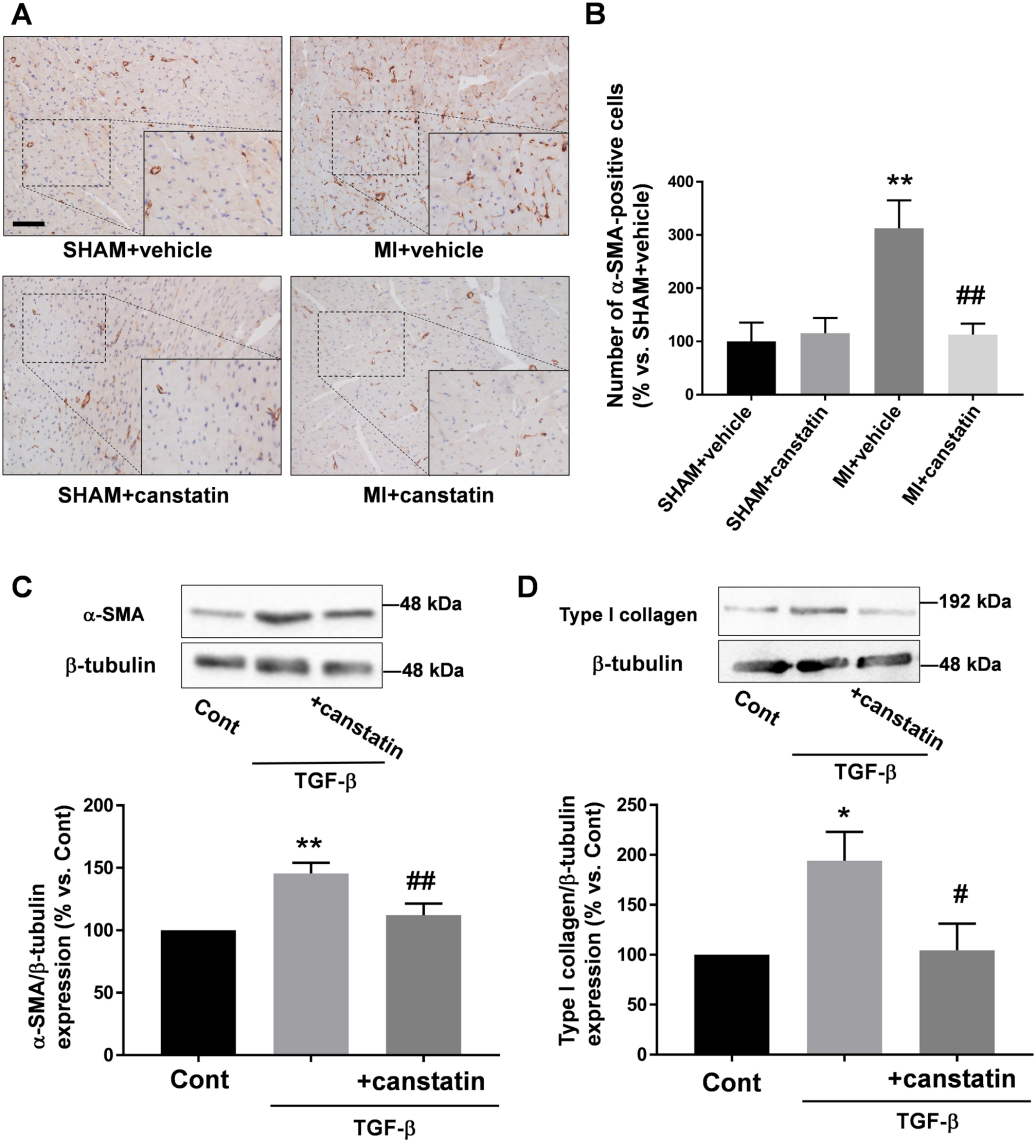
Canstatin prevents increase of myofibroblasts in non-infarcted area after MI and suppresses transforming growth factor (TGF)-β1 -induced differentiation of cardiac fibroblasts into myofibroblasts. (**A, B**) Recombinant mouse canstatin (20 μg/kg) or vehicle was intraperitoneally administered for 28 days after LAD ligation. The hearts were isolated and then thin paraffin Sects. (4 μm) were made. (**A**) Representative pictures of the non-infarcted areas from SHAM + vehicle, SHAM + canstatin, MI + vehicle and MI + canstatin groups reacted with a specific antibody against α-smooth muscle actin (α-SMA) were shown. Scale bar: 100 μm. (**B**) The number of myofibroblasts, a non-vascular α-SMA-positive cell, in 3 fields was counted, and the normalized number relative to SHAM + vehicle was shown as mean ± S.E.M. (SHAM + vehicle and SHAM + canstatin: n = 6; MI + vehicle and MI + canstatin: n = 8). **P < 0.01 vs. SHAM + vehicle, ## P < 0.01 vs. MI + vehicle (two-way ANOVA followed by Tukey’s post hoc test). (**C**) Cardiac fibroblasts isolated from ventricles of normal rats were stimulated with TGF-β1 (10 ng/ml) for 48 h in the presence or absence of recombinant mouse canstatin (250 ng/ml, 30 min pre-treatment), and the cell lysates were collected. Expressions of α-SMA (C) and type I collagen (D) were detected by Western blotting. (**Upper**) Representative blots of α-SMA, type I collagen and β-tubulin were shown. (**Lower**) Levels of α-SMA and type I collagen were corrected by β-tubulin, and the normalized expressions relative to Cont were shown as mean ± S.E.M. (C: n = 7; D: n = 5). *, **P < 0.05, 0.01 vs. Cont, #, ## P < 0.05, 0.01 vs. TGF-β (one-way ANOVA followed by Tukey’s post hoc test).

During the development of adverse cardiac remodeling, cardiac fibroblasts differentiate into myofibroblasts, which highly express α-SMA and type I collagen, a main constituent for interstitial fibrosis^9^. We examined the effects of canstatin on the differentiation by immunohistochemical staining (SHAM + vehicle and SHAM + canstatin: n = 6, MI + vehicle and MI + canstatin: n = 8, Fig. 7A). MI significantly increased α-SMA-positive myofibroblasts in non-infarcted area, which was significantly prevented by canstatin (MI + vehicle: 312 ± 53% vs. SHAM + vehicle, P < 0.01; MI + canstatin: 113 ± 21%, P < 0.01 vs. MI + vehicle, Fig. 7B). Canstatin-alone administration had no effect on the number of myofibroblasts (SHAM + canstatin: 115 ± 29%, Fig. 7B).

We also performed in vitro experiments to clarify the direct effects of canstatin on transforming growth factor (TGF)-β1-induced differentiation of cardiac fibroblasts by measuring expression of α-SMA (n = 7, Fig. 7C) and type I collagen (n = 5, Fig. 7D). TGF-β1 significantly increased the expression of α-SMA and type I collagen (α-SMA; TGF-β: 146 ± 8% vs. Cont, P < 0.01, type I collagen; TGF-β: 194 ± 29% vs. Cont, P < 0.05). Recombinant mouse canstatin (250 ng/ml, 30 min pre-treatment) significantly prevented it (α-SMA; TGF-β + canstatin: 112 ± 9%, P < 0.01 vs. TGF-β, type I collagen; TGF-β + canstatin: 105 ± 27%, P < 0.05 vs. TGF-β).

## Discussion

The present study for the first time demonstrated that recombinant mouse canstatin improved survival rate and suppressed adverse cardiac remodeling, such as LV dilatation, scar thinning in the infarcted area as well as cardiac hypertrophy and interstitial fibrosis in the non-infarcted area after MI.

We previously examined the effects of canstatin (10–250 ng/ml) on hypoxia- or isoproterenol-induced apoptosis in H9c2 rat cardiomyoblasts and found that 250 ng/ml of canstatin significantly inhibited apoptosis^11,17^. According to the estimated blood volume/body weight (64 ml/kg), the injection of recombinant canstatin (20 μg/kg) may reach approximately 310 ng/ml as a blood concentration, which can exert cardioprotective effects. In addition, we previously demonstrated that the administration of recombinant canstatin (4, 20 and 40 μg/kg) to isoproterenol-induced cardiac hypertrophy model rats inhibited the increase of LV weight in a dose-dependent manner^14^. Although the minimum dose (4 μg/kg) of canstatin did not significantly inhibit the increase of LV weight, the middle dose (20 μg/kg) significantly inhibit it. From these observations, we used 20 μg/kg of canstatin in the present study.

In the present study, canstatin improved survival rate after MI. The protective effect of canstatin was observed within 9 days after MI (52.9% of rats in MI + vehicle group died within 7 days after MI and 27.3% of the rats in MI + canstatin group died within 9 days) (Fig. 1). In addition, canstatin significantly suppressed the increase of LVID and improved FS and EF within 7 days (Fig. 2). From these results, it was suggested that canstatin improved myocardium injury in the early phase of MI. We previously reported that canstatin inhibited hypoxia-induced apoptosis in H9c2 rat cardiomyoblasts^11^. Thus, we examined whether canstatin exerts cytoprotective effect against acute ischemic stress by an ex vivo MI model. A knockdown of canstatin protein achieved by injecting small interference RNA of type IV collagen α2 chain gene exaggerated an infarct size and apoptosis of cardiomyocytes after left anterior descending artery (LAD) ligation (Fig. S1). Therefore, it is assumed that canstatin might improve survival rate, LV dilatation and dysfunction in the early phase of MI perhaps in part through the inhibition of apoptosis of cardiomyocytes. Further studies are needed to clarify the importance of early phase treatment of canstatin in MI.

In the present study, canstatin significantly thickened the scar tissue after MI (Fig. 3). Scar tissue is mainly composed of collagens (type I and III), which are produced by myofibroblasts differentiated from cardiac fibroblasts^3^. An insufficiency of the population and activation of myofibroblasts in infarcted area leads to the formation of fragile scar tissue, which results in systolic dysfunction, adverse cardiac remodeling and cardiac rupture^15^. We previously demonstrated that canstatin promoted migration of cardiac fibroblasts and proliferation of myofibroblasts derived from the infarcted area after MI^12,18^. Therefore, it was suggested that canstatin might prevent scar thinning by promoting adequate scar formation via regulating the functions of cardiac fibroblasts and myofibroblasts in the infarcted area.

In the present study, canstatin inhibited cardiac hypertrophy in the non-infarcted area after MI (Fig. 4). We previously reported that recombinant mouse canstatin inhibited isoproterenol-induced cardiac hypertrophy through the suppression of intracellular Ca^2+^ rise and subsequent activation of calcineurin/nuclear translocation of NFATc4^14^. We also demonstrated that canstatin suppressed L-type Ca^2+^ channel activity in rat cardiomyocytes^19^. Sanchez-Alonso et al. reported that the open probability of L-type Ca^2+^ channel on surface of cardiomyocytes in MI-induced heart failure model rats increased^20^. In addition, it has been reported that the phosphatase activity of calcineurin and the transcriptional activity of NFAT were increased in the heart tissue of MI model mice and rats, respectively^21,22^. The present study showed that canstatin inhibited the nuclear translocation of NFATc4 in cardiomyocytes of the non-infarcted area (Fig. 5). Calcineurin activated by intracellular Ca^2+^ rise induces dephosphorylation and nuclear translocation of NFATc4, which promotes transcription of hypertrophy-related genes^16,23^. Thus, canstatin might inhibit cardiac hypertrophy in the non-infarcted area perhaps in part through the suppression of Ca^2+^/calcineurin/NFATc4 pathway after MI. The limitation of this study was that we were not able to determine precise inhibitory mechanisms of canstatin on cardiac hypertrophy of the non-infarcted area in MI model. Canstatin might affect other hypertrophic pathways, such as activation of calmodulin-dependent protein kinase II and increase of reactive oxygen species, which are induced by an intracellular Ca^2+^ rise^24,25^. Further studies are needed to clarify the detailed mechanisms of canstatin for the inhibition of cardiac hypertrophy after MI.

In the present study, canstatin inhibited the interstitial fibrosis in the non-infarcted area (**Fig. ****6**). Interstitial fibrosis is an adverse cardiac remodeling since it increases stiffness of myocardium which leads to cardiac dysfunction^9^. TGF-β synthesized and secreted after MI is a major cause for developing interstitial fibrosis in the non-infarcted area^9^. Mechanical stress in the non-infarcted area activates latent TGF-β, which promotes differentiation of cardiac fibroblasts into myofibroblasts^9^. In the present study, the number of α-SMA-positive myofibroblasts was decreased by canstatin in the non-infarcted area. Thus, it was suggested that canstatin might inhibit interstitial fibrosis in the non-infarcted area through the inhibition of differentiation of cardiac fibroblasts into myofibroblasts. In the present study, canstatin significantly inhibited TGF-β1-induced α-SMA expression in cultured cardiac fibroblasts (Fig. 7). α_v_β_3_ and α_v_β_5_ integrins are thought to be functional receptors for canstatin^11,26^. It has been reported that cilengitide, an inhibitor of α_v_β_3_ and α_v_β_5_ integrins, attenuated TGF-β1-induced differentiation of cardiac fibroblasts into myofibroblasts^27^. In addition, latent TGF-β1 was activated via α_v_β_3_ and α_v_β_5_ integrins in response to mechanical stretch of fibroblasts/myofibroblasts^28,29^. We previously demonstrated that canstatin inhibited collagen gel contraction by cardiac myofibroblasts^12^. Therefore, canstatin might inhibit TGF-β1-induced differentiation of cardiac fibroblasts into myofibroblasts by regulating α_v_β_3_ and α_v_β_5_ integrins and/or cellular contraction. Further study is needed to clarify the detailed inhibitory mechanism of canstatin in differentiation of fibroblasts.

Canstatin is known to be a potent anti-angiogenic factor^10^. Thus, we examined whether canstatin affects angiogenesis by immunohistochemical staining against antibody to CD31, an endothelial cell marker. We found that canstatin had no effect on the number of CD31-positive capillary vessels in non-infarcted area (Fig. S2), suggesting that canstatin had no effect on angiogenesis after MI. It has been reported that recombinant canstatin-treatment in the range of 3–10 mg/kg exerted anti-tumor effects through the inhibition of angiogenesis in tumor model mice^10,30^. Thus, canstatin at the lower concentration (20 μg/kg) used in this study might have no effect on angiogenesis. Inflammation in non-infarcted area after MI is involved in LV remodeling^31^. Tumor necrosis factor (TNF)-α and interleukin (IL)-6, an inflammatory cytokine, were upregulated in non-infarcted area after MI^32^. In the present study, we found that canstatin had no significant effects on the increase in mRNA expression of TNF-α and IL-6 mediated by MI (Data not shown, SHAM + vehicle and SHAM + canstatin: n = 6; MI + vehicle and MI + canstatin: n = 8). Therefore, the cardioprotective effect of canstatin might not be due to the inhibition of inflammation.

In conclusion, our study for the first time demonstrated that canstatin is a novel endogenous peptide, which prevents adverse cardiac remodeling after MI. MI remains the most common cause of heart failure throughout the world^1,2^. The present study thus provides an insight into the development of a novel therapeutic strategy to improve prognosis of MI-induced dysfunction leading to heart failure.

## Methods

### Reagent and antibodies

Reagents sources were as follows: recombinant mouse canstatin (produced by Escherichia coli as described previously)^14^ and TGF-β1 (PeproTech, Rocky Hill, NJ, U.S.A).

Antibody sources were as follows: anti-NFATc4 antibody (Santa Cruz Biotechnology, Santa Cruz, CA, U.S.A.), anti-type I collagen antibody (Rockland Immunochemicals, Gilbertsville, PA, U.S.A), anti-α-SMA antibody (Dako, Glostrup, Denmark), anti-β-tubulin antibody (Wako, Osaka, Japan), anti-rabbit IgG horseradish peroxidase linked whole antibody and anti-mouse IgG horseradish peroxidase linked whole antibody (Cell signaling Technology, Danvers, MA, U.S.A.).

### MI model

All animal experiments were approved by the President of Kitasato University through the judgment by Institutional Animal Care and Use Committee of Kitasato University (Approval No. 17-082, 18-019, 19-126). Male Wistar rats (CLEA Japan, Tokyo, Japan) were cared in accordance with the guideline for animal care and treatment of the Kitasato University. MI was induced by ligating coronary artery of rats (7–8-week-old) as described previously^12,13^. Rats were anesthetized with isoflurane (induction: 5%, maintenance: 2.5%) and ventilated (respiratory rate: 100 times/min, tidal volume: 5 ml) through an intubation tube. Buprenorphine (0.005 mg/100 g) was subcutaneously administered for an analgesia. After left thoracotomy was performed, LAD was permanently ligated using a 6–0 nylon suture and the chest was closed. LAD ligation was not performed in SHAM-operated rats. Recombinant mouse canstatin (20 μg/kg) or its vehicle (0.8 mM Tris, 20 mM L-Arginine, 4% glycerol in saline) was intraperitoneally administered for 28 days from the day of operation. Twenty eight days after the operation, the heart was isolated and perfused with Krebs–Henseleit solution (119 mM NaCl, 4.8 mM KCl, 2.5 mM CaCl_2_, 1.2 mM KH_2_PO_4_, 1.2 mM MgSO_4_, 24.9 mM NaHCO_3_, 10.0 mM glucose). A part of isolated ventricular tissue of the heart was separated into infarcted area and non-infarcted area, which were immediately frozen with liquid nitrogen and preserved at – 80 °C for Western blotting. Remaining ventricular tissue of the heart was cut transversely and fixed with 10% neutral buffered formalin for histological analysis.

### Echocardiography

Echocardiography was performed at 3, 7, 14, 21 and 28 days after MI by using SonoScape X5V (SonoScape Medical Corp., Shenzhen, China) as described previously^14,33^. The rats were anesthetized with 2–3% isoflurane and properly positioned. M-mode images at longitudinal axis between papillary muscles were obtained from a parasternal short axis view of LV. LVIDd, LVIDs, FS and EF were measured from the images.

### HE staining

HE staining was performed to measure the cell size of cardiomyocytes as described previously^34^. LV tissues fixed with 10% neutral buffered formalin were embedded in paraffin and sectioned (4 μm). The deparaffinized sections were incubated with hematoxylin for 5 min. Following a rinse with distilled water, the sections were stained with eosin for 30 min. The images were obtained using a light microscope (BX-51; OLYMPUS, Tokyo, Japan) equipped with a microscope digital camera (DP74; OLYMPUS). The diameter of 50 cardiomyocytes in non-infarcted area of left ventricle was measured by cellSens Imaging Software (OLYMPUS) in each rat.

### Picrosirius red staining

Picrosirius red staining was performed to evaluate scar formation in the infarcted area and fibrosis of the non-infarcted area as described previously^34^. LV tissues fixed with 10% neutral buffered formalin were embedded in paraffin and sectioned (4 μm). The deparaffinized sections were stained with iron hematoxylin solution for 8 min and stained with picrosirius red solution for 60 min. The images were obtained using a light microscope (BX-51) equipped with a microscope digital camera (DP74). Scar thickness was measured by cellSens Imaging Software (OLYMPUS) in the randomly selected fifteen points of the scar tissue. The fibrosis of non-infarcted area in left ventricle was measured by Image J software (National Institutes of Health, Bethesda, MD, USA) in three high power fields, and fibrotic area (in %) (picrosirius red stained area/total area) was calculated.

### Immunohistochemical staining

Immunohistochemical staining was performed to evaluate nuclear translocation of NFATc4 as described previously^14^. LV tissues fixed with 10% neutral buffered formalin were embedded in paraffin and sectioned (4 μm). The deparaffinized sections were heated with a microwave for antigen retrieval in Tris-ethylenediaminetetraacetic acid (EDTA) buffer (pH 9.0). The sections were incubated in methanol with 0.3% H_2_O_2_ for 20 min to block endogenous peroxidase activity, and then incubated with anti-NFATc4 antibody (1:100 dilution) at 4 °C overnight. After washing, the sections were incubated in biotinylated link (Dako) for 60 min and next in streptavidin–horseradish peroxidase (Dako) for 30 min at room temperature. Then, antigen–antibody reaction was visualized by a liquid DAB + substrate chromogen system (Dako). The nuclei were counterstained with hematoxylin. The images were obtained using a light microscope (BX-51) equipped with a microscope digital camera (DP74). The NFATc4 positive nuclei were counted in three high power fields, and NFATc4-positive nuclei/total nuclei was calculated.

### Isolation and culture of cardiac fibroblasts

Cardiac fibroblasts were isolated from the ventricle of male Wistar rats (4–5-week-old) and cultured as described previously^35^. The isolated heart was perfused with 0.02% collagenase (Wako) by a Langendorff apparatus. Then, the ventricle was minced and suspended in Dulbecco’s modified Eagle’s medium (DMEM, Wako). After centrifugation, the suspended cells were dispersed in DMEM containing 10% fetal bovine serum (FBS; Gibco, Carlsbad, CA, U.S.A.) and 1% antibiotic–antimycotic mixed solution (Nacalai Tesque, Kyoto, Japan) on 2% gelatin-coated culture dish and incubated for 90 min at 37 °C in 5% CO_2_. After incubation, the floating cells were removed and the adhered cells were cultured in DMEM containing 10% FBS and 1% antibiotic–antimycotic mixed solution. The cells (passage 1–2) were starved for 24 h in DMEM before treatment. Differentiation of cardiac fibroblasts into myofibroblasts was induced by a stimulation of human recombinant TGF-β1 (10 ng/ml) for 48 h. Recombinant canstatin was treated 30 min before the TGF-β1 stimulation.

### Western blotting

Western blotting was performed as described previously^14^. The isolated LV tissues of non-infarcted were homogenized with Cell destroyer (Bio Medical Science Inc., Tokyo, Japan), and total protein of the tissues was extracted by RIPA lysis buffer (20 mM Tris–HCl pH 7.4, NaCl 150 mM, MgCl_2_ 10 mM, 1% TritonX-100, 0.1% sodium dodecyl sulfate, 0.5% sodium deoxycholate) containing 1% protease inhibitor cocktail (Nacalai Tesque). Total protein of cardiac fibroblasts was extracted by cell lysis buffer (Cell signaling Technology) containing 1% protease inhibitor cocktail (Nacalai Tesque). Sodium dodecyl sulfate–polyacrylamide gel electrophoresis was performed to separate equal amounts of proteins (LV tissue lysate: 20 μg, cell lysate: 1 or 10 μg), and the proteins were transferred to a nitrocellulose membrane (Pall Corporation, Ann Arbor, MI, U.S.A.). The membranes were incubated in 0.5% skim milk for blocking non-specific binding of antibody to antigen. After overnight incubation with the primary antibody at 4 °C, the membranes were incubated with secondary antibody. The chemiluminescent signal was detected by EZ-ECL detecting reagents (Biological Industries, Kibbutz Beit, Haemek, Israel) using an ATTO light capture system (AE-6972; ATTO, Tokyo, Japan). The images of chemiluminescent signals were analyzed with a CS Analyzer 3.0 software (ATTO).

### Statistical analysis

The results were presented as mean ± standard error of the mean (S.E.M.). In two-group comparison, statistical analyses were performed by unpaired two-tailed Student’s *t* test (Fig. 3B). In multi-group comparison, statistical analyses were performed by one-way (Fig. 7C–D) or two-way (Figs. 2B–E, 4B, 5B, 6B, C, 7B) ANOVA followed by Tukey’s post hoc test. In the survival study, statistical analysis was performed by log-rank test (Fig. 1). A value of P < 0.05 was considered statistically significant.

## Supplementary information

Supplementary information
