# Supplementary material for: Long-term administration of recombinant canstatin prevents adverse cardiac remodeling after myocardial infarction
Source: Sci Rep. 2020 Jul 30;10:12881. doi: 10.1038/s41598-020-69736-y (PMC7393096; doi:10.1038/s41598-020-69736-y)
Supplement: Supplementary file 1 — Supplementary information [file 41598_2020_69736_MOESM1_ESM.pdf]

Long-term administration of recombinant canstatin prevents adverse cardiac remodeling after myocardial infarction

Akira Sugiyama[1], Rumi Ito[1], Muneyoshi Okada[1]*, and Hideyuki Yamawaki[1]

[1]*Laboratory of Veterinary Pharmacology, School of Veterinary Medicine, Kitasato University, Towada city, Aomori, 0348628, Japan*

*CORRESPONDENCE TO: Muneyoshi Okada, D.V.M., Ph.D.
Laboratory of Veterinary Pharmacology,
School of Veterinary Medicine, Kitasato University, Higashi 23 bancho 35-1, Towada city,
Aomori 034-8628, Japan
Phone: +81-176-23-4371; FAX: +81-176-23-8703
E-mail address: mokada@vmas.kitasato-u.ac.jp

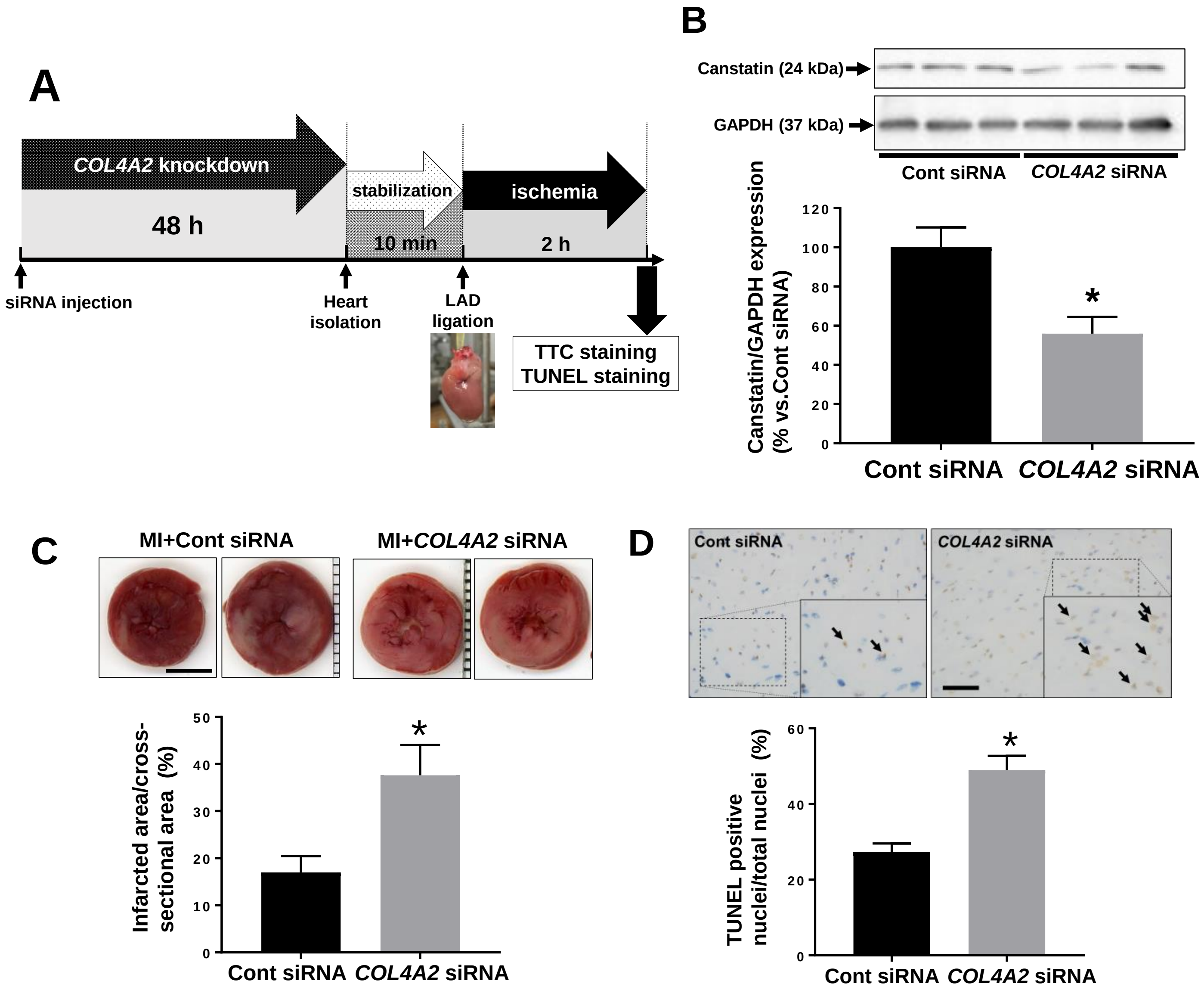

Supplementary fig. 1. Canstatin knockdown by injecting small interference RNA (siRNA) of *COL4A2* (type IV collagen α2 chain gene) enhances *ex vivo* myocardial infarction induced by coronary arterial ligation in rats. (**A**) Twenty microgram of *COL4A2* siRNA or Control (Cont) siRNA was injected in male Wistar rats (7-8-week-old) via jugular vein as described previously [1]. Forty eight hours after siRNA-injection, the heart was isolated and perfused with oxygenated normal 2-[4-(2-Hydroxyethl)-1-piperazinyl] ethanesulfonic acid (HEPES)-Tyrode solution (NaCl 143 mM, KCl 5.4 mM, $NaH_2PO_4 \cdot 2H_2O$ 0.33 mM, $MgCl_2 \cdot 6H_2O$ 0.5 mM, Glucose 5.5 mM, HEPES 5 mM, $CaCl_2$ 1.8 mM: pH 7.4) using a Langendorff apparatus. Ten minutes after stabilization, left anterior descending artery (LAD) was ligated by 6-0 nylon. Two hours after LAD ligation, the heart was isolated. (**B**) Suppression of canstatin protein in infarcted area of the LAD-ligated heart tissue by *COL4A2* siRNA-injection was confirmed by Western blotting. (**Upper**) Representative blots for canstatin (24 kDa) and glyceraldehyde-3-phosphate dehydrogenase (GAPDH) (37 kDa) of Cont siRNA group and *COL4A2* siRNA group were shown. (**Lower**) Levels of canstatin protein expression were corrected by GAPDH, and the normalized expression relative to Cont siRNA was shown as means±standard error of the mean

(S.E.M.) (Cont siRNA: n=3; *COL4A2* siRNA: n=3) *P<0.05 vs. Cont siRNA (two-tailed Student's *t* test). (**C**) Cross-section of the LAD-ligated heart was incubated in 1% 2,3,5-triphenyl tetrazolium chloride (TTC) solution for 15 min at 37 ºC. (**Upper**) Representative TTC-stained sections for the heart of Cont siRNA group and *COL4A2* siRNA group were shown. The viable tissue (non-infarcted area) was stained red, while the infarcted area was stained white. Scale bar: 5 mm. (**Lower**) The ratio of infarcted area/total cross-sectional area (in %) was calculated and shown as means±S.E.M. (Cont siRNA: n=4; *COL4A2* siRNA: n=4) * P<0.05 vs Cont siRNA (two-tailed Student's *t* test). (**D**) The heart tissue was embedded in optimal cutting temperature compound and the frozen sections (6 μm) were made. Terminal deoxynucleotidyl transferase dUTP nick end labeling (TUNEL) staining for the sections was performed to detect apoptosis. The nuclei were counterstained with hematoxylin. (**Upper**) Representative pictures for the infarcted area stained with TUNEL of Cont siRNA group and *COL4A2* siRNA group were shown. Scale bar: 50 μm. (**Lower**) The TUNEL-positive nuclei were counted in three high power fields. The ratio of TUNEL-positive nuclei/total nuclei (%) was calculated and shown as means±S.E.M. (Cont siRNA: n=3; *COL4A2* siRNA: n=4). * P<0.05 vs. Cont siRNA (two-tailed Student's *t* test).

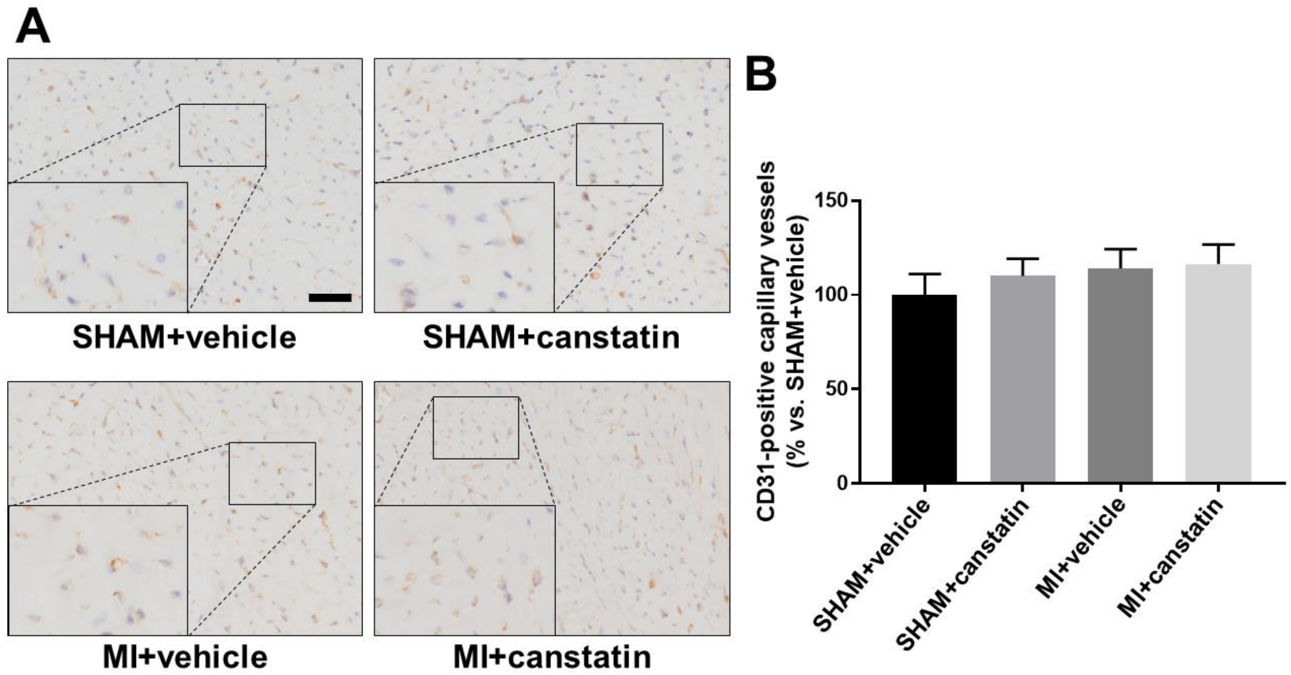

Supplementary fig. 2. Canstatin had no effect on angiogenesis in non-infarcted area after MI. (**A**) Recombinant mouse canstatin (20 μg/kg) or vehicle was intraperitoneally administered for 28 days after LAD ligation in rats. The hearts were isolated and then thin paraffin sections (4 μm) were made. Representative pictures of the non-infarcted areas from SHAM-operated (SHAM)+vehicle, SHAM+canstatin, MI+vehicle and MI+canstatin groups reacted with a specific antibody against CD31 were shown. The nuclei were counterstained with hematoxylin. Scale bar: 50 μm. (B) The number of CD31-positive capillary vessels in 3 fields was counted, and the normalized number relative to SHAM+vehicle was shown as mean±S.E.M. (SHAM+vehicle and SHAM+canstatin: n=6; MI+vehicle and MI+canstatin: n=8, two-way ANOVA followed by Tukey's post hoc test).
